# 2,2-Dimethyl-2,3-dihydro-1-benzofuran-7-yl *N*-ethyl­carbamate

**DOI:** 10.1107/S1600536809044687

**Published:** 2009-10-31

**Authors:** Wen-Sheng Li, Li Li, Jiang-Sheng Li

**Affiliations:** a College of Chemistry and Chemical Engineering, Hunan University, Changsha 410082, People’s Republic of China; b School of Chemistry & Biological Engineering, Changsha University of Science & Technology, Changsha 410004, People’s Republic of China

## Abstract

The title compound, C_13_H_17_NO_3_, crystallizes with two independent mol­ecules in the asymmetric unit. In the crystal, N—H⋯O hydrogen bonds link the mol­ecules, forming chains propagaiting in [100]. A weak C—H⋯O inter­action also occurs.

## Related literature

For background on insecticides related to the title compound, see: Tomlin (1994 ▶). For a related structure, see Xu *et al.* (2005 ▶).
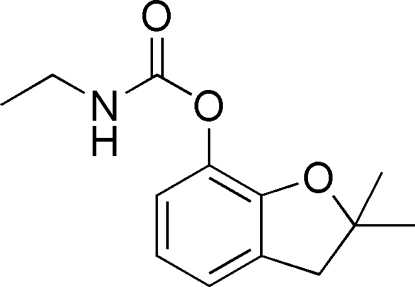


## Experimental

### 

#### Crystal data

C_13_H_17_NO_3_
*M* *_r_* = 235.28
Orthorhombic, 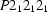

*a* = 10.362 (2) Å
*b* = 13.962 (3) Å
*c* = 18.069 (4) Å
*V* = 2614.1 (10) Å^3^
*Z* = 8
Mo *K*α radiation
μ = 0.09 mm^−1^
*T* = 293 K
0.26 × 0.20 × 0.08 mm

#### Data collection

Rigaku Saturn CCD area-detector diffractometer
Absorption correction: multi-scan (*CrystalClear*; Rigaku/MSC, 2005 ▶) *T*_min_ = 0.978, *T*_max_ = 0.993
20967 measured reflections
3256 independent reflections
2338 reflections with *I* > 2σ(*I*)
*R*_int_ = 0.049

#### Refinement

*R*[*F* ^2^ > 2σ(*F* ^2^)] = 0.056
*wR*(*F* ^2^) = 0.154
*S* = 1.08
3256 reflections
322 parameters
H atoms treated by a mixture of independent and constrained refinement
Δρ_max_ = 0.14 e Å^−3^
Δρ_min_ = −0.14 e Å^−3^

### 

Data collection: *CrystalClear* (Rigaku/MSC, 2005 ▶); cell refinement: *CrystalClear*; data reduction: *CrystalClear*; program(s) used to solve structure: *SHELXS97* (Sheldrick, 2008 ▶); program(s) used to refine structure: *SHELXL97* (Sheldrick, 2008 ▶); molecular graphics: *SHELXTL* (Sheldrick, 2008 ▶); software used to prepare material for publication: *SHELXL97*.

## Supplementary Material

Crystal structure: contains datablocks I, global. DOI: 10.1107/S1600536809044687/hb5190sup1.cif

Structure factors: contains datablocks I. DOI: 10.1107/S1600536809044687/hb5190Isup2.hkl

Additional supplementary materials:  crystallographic information; 3D view; checkCIF report

## Figures and Tables

**Table 1:** Hydrogen-bond geometry (Å, °)

*D*—H⋯*A*	*D*—H	H⋯*A*	*D*⋯*A*	*D*—H⋯*A*
N1—H1⋯O6	0.82 (4)	2.28 (4)	3.024 (4)	151 (3)
N2—H2⋯O2^i^	0.84 (4)	2.19 (4)	2.985 (4)	156 (3)
C19—H19⋯O5^ii^	0.93	2.48	3.269 (4)	143

Symmetry codes: (i) 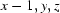; (ii) 
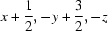
.

**Fig. 1.**
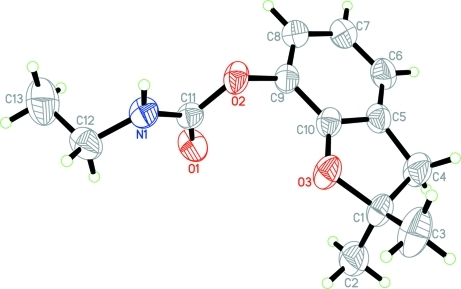
One molecule in the asymmetric unit of (I), showing d isplacement ellipsoids drawn at the 30% probability level.

**Fig. 2.**
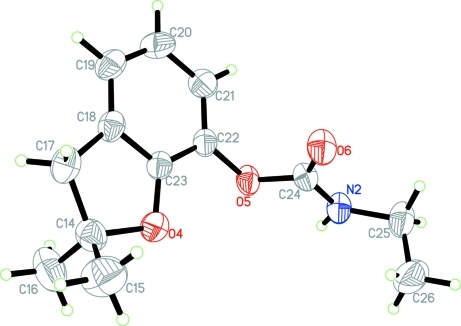
The other molecule in the asymmetric unit of (I), showing displacement ellipsoids drawn at the 30% level.

## supplementary crystallographic information

### Comment

The title compound, (I), is an anologue to commercial Carbofuran, which is a popular carbamate insecticide (Tomlin, 1994). Herein, we present its single-crystal structure: it crystallizes with two independent molecules in the asymmetric unit (Figs. 1 & 2), and it has the same space group P212121 like Carbofuran reported previouly (Xu *et al.*, 2005). In the molecule shown in Fig 1, the dihedral angle between the carbamate plane O1/C11/O2/N1 and the benzo ring C5—C10 is 78.50 (5)°, and atom C1 deviates from the C4—C10/O3 plane with an angle of 0.167 (2) Å. In the other molecule, shown in Fig 2, the dihedral angle between the plane O5/O6/C24/N2 and the benzo ring C18—C23 is 79.87 (5)°, and atom C14 lies 0.175 (2)Å out of the plane C17—C23/O4. All these are similar to those reported in the literature (Xu *et al.*, 2005).

In the crystal structure, the two independent molecules in the asymmetric unit are linked by a strong N—H···O hydrogen bond, and each links another adjacent molecule by the N—H···O hydrogen bond. Besides, weak C—H···O H-bonding consolidates the packing (Table 1).

### Experimental

The title compound was prepared by reaction of 2,3-dihydro-7-hydroxy-2,2-dimethylbenzofuran with ethylcarbamoyl chloride in 283 K, with a 85% yeild.  Colourless prisms of (I) were obtained by evaporation from its ethanoic solution at room temperature.

### Refinement

All C-bound H atoms were positioned geometrically and constrained to ride on their parent atoms [C—H distances are 0.93 and 0.97Å with *U*_iso_(H) = 1.2 *U*_eq_(C) for aromatic and CH_2_ H atoms, 0.96Å with *U*_iso_ = 1.5*U*_eq_ (C) for CH_3_ H atoms].

The position and isotropic displacement parameters of the NH H atoms were refined freely. In the absence of significant anomalous dispersion effects, Friedel pairs were merged.

### Crystal data

**Table:**

C_13_H_17_NO_3_	*F*(000) = 1008
*M**_r_* = 235.28	*D*_x_ = 1.196 Mg m^−^^3^
Orthorhombic, *P*2_1_2_1_2_1_	Mo *K*α radiation, λ = 0.71073 Å
Hall symbol: P 2ac 2ab	Cell parameters from 5268 reflections
*a* = 10.362 (2) Å	θ = 1.8–27.1°
*b* = 13.962 (3) Å	µ = 0.09 mm^−^^1^
*c* = 18.069 (4) Å	*T* = 293 K
*V* = 2614.1 (10) Å^3^	Prism, colourless
*Z* = 8	0.26 × 0.20 × 0.08 mm

### Data collection

**Table:**

Rigaku Saturn CCD area-detector diffractometer	3256 independent reflections
Radiation source: rotating anode	2338 reflections with *I* > 2σ(*I*)
confocal	*R*_int_ = 0.049
Detector resolution: 7.31 pixels mm^-1^	θ_max_ = 27.2°, θ_min_ = 1.8°
ω and φ scans	*h* = −10→13
Absorption correction: multi-scan (*CrystalClear*; Rigaku/MSC, 2005)	*k* = −17→17
*T*_min_ = 0.978, *T*_max_ = 0.993	*l* = −21→23
20967 measured reflections	

### Refinement

**Table:**

Refinement on *F*^2^	Secondary atom site location: difference Fourier map
Least-squares matrix: full	Hydrogen site location: inferred from neighbouring sites
*R*[*F*^2^ > 2σ(*F*^2^)] = 0.056	H atoms treated by a mixture of independent and constrained refinement
*wR*(*F*^2^) = 0.154	*w* = 1/[σ^2^(*F*_o_^2^) + (0.083*P*)^2^] where *P* = (*F*_o_^2^ + 2*F*_c_^2^)/3
*S* = 1.08	(Δ/σ)_max_ = 0.001
3256 reflections	Δρ_max_ = 0.14 e Å^−^^3^
322 parameters	Δρ_min_ = −0.14 e Å^−^^3^
0 restraints	Extinction correction: *SHELXL97* (Sheldrick, 2008), Fc^*^=kFc[1+0.001xFc^2^λ^3^/sin(2θ)]^-1/4^
Primary atom site location: structure-invariant direct methods	Extinction coefficient: 0.029 (3)

### Special details

**Table:**

Geometry. All e.s.d.'s (except the e.s.d. in the dihedral angle between two l.s. planes) are estimated using the full covariance matrix. The cell e.s.d.'s are taken into account individually in the estimation of e.s.d.'s in distances, angles and torsion angles; correlations between e.s.d.'s in cell parameters are only used when they are defined by crystal symmetry. An approximate (isotropic) treatment of cell e.s.d.'s is used for estimating e.s.d.'s involving l.s. planes.
Refinement. Refinement of *F*^2^ against ALL reflections. The weighted *R*-factor *wR* and goodness of fit *S* are based on *F*^2^, conventional *R*-factors *R* are based on *F*, with *F* set to zero for negative *F*^2^. The threshold expression of *F*^2^ > σ(*F*^2^) is used only for calculating *R*-factors(gt) *etc*. and is not relevant to the choice of reflections for refinement. *R*-factors based on *F*^2^ are statistically about twice as large as those based on *F*, and *R*- factors based on ALL data will be even larger.

### Fractional atomic coordinates and isotropic or equivalent isotropic displacement parameters (Å^2^)

**Table:**

	*x*	*y*	*z*	*U*_iso_*/*U*_eq_	
O1	0.9284 (3)	0.3257 (2)	0.01889 (15)	0.0801 (8)	
O2	0.9054 (2)	0.50324 (18)	0.14502 (19)	0.0855 (9)	
O3	0.7620 (2)	0.38179 (17)	0.13896 (15)	0.0725 (7)	
O4	0.3318 (3)	0.79075 (16)	0.11613 (14)	0.0758 (7)	
O5	0.2451 (2)	0.60024 (16)	0.08020 (14)	0.0688 (7)	
O6	0.4027 (2)	0.51258 (19)	0.13447 (16)	0.0812 (8)	
N1	0.6932 (3)	0.5302 (2)	0.12561 (19)	0.0709 (8)	
N2	0.1916 (3)	0.4821 (2)	0.15411 (17)	0.0660 (8)	
C1	1.0315 (4)	0.2815 (3)	−0.0269 (2)	0.0762 (10)	
C2	1.1341 (5)	0.3563 (3)	−0.0360 (3)	0.1080 (16)	
H2A	1.0989	0.4107	−0.0614	0.162*	
H2B	1.2044	0.3303	−0.0641	0.162*	
H2C	1.1647	0.3757	0.0119	0.162*	
C3	0.9693 (6)	0.2508 (4)	−0.0979 (3)	0.127 (2)	
H3A	0.9036	0.2043	−0.0875	0.190*	
H3B	1.0332	0.2232	−0.1298	0.190*	
H3C	0.9312	0.3054	−0.1217	0.190*	
C4	1.0807 (5)	0.1960 (3)	0.0181 (2)	0.0931 (13)	
H4A	1.0517	0.1360	−0.0033	0.112*	
H4B	1.1743	0.1958	0.0205	0.112*	
C5	1.0226 (4)	0.2114 (2)	0.0936 (2)	0.0729 (10)	
C6	1.0378 (5)	0.1657 (3)	0.1605 (3)	0.0881 (12)	
H6	1.0972	0.1161	0.1652	0.106*	
C7	0.9655 (5)	0.1933 (3)	0.2198 (3)	0.0947 (13)	
H7	0.9766	0.1626	0.2650	0.114*	
C8	0.8759 (4)	0.2665 (3)	0.2138 (2)	0.0840 (12)	
H8	0.8262	0.2840	0.2545	0.101*	
C9	0.8606 (3)	0.3134 (2)	0.1472 (2)	0.0679 (9)	
C10	0.9355 (3)	0.2866 (3)	0.0874 (2)	0.0662 (9)	
C11	0.7964 (3)	0.4767 (2)	0.13721 (19)	0.0612 (8)	
C12	0.6965 (4)	0.6335 (3)	0.1279 (3)	0.0935 (13)	
H12A	0.6404	0.6590	0.0897	0.112*	
H12B	0.7836	0.6554	0.1179	0.112*	
C13	0.6545 (7)	0.6699 (4)	0.2009 (4)	0.136 (2)	
H13A	0.5680	0.6489	0.2107	0.205*	
H13B	0.6571	0.7387	0.2008	0.205*	
H13C	0.7111	0.6459	0.2386	0.205*	
C14	0.3854 (4)	0.8896 (3)	0.1104 (2)	0.0773 (10)	
C15	0.4434 (6)	0.9120 (5)	0.1854 (3)	0.1229 (18)	
H15A	0.3771	0.9094	0.2225	0.184*	
H15B	0.4806	0.9749	0.1845	0.184*	
H15C	0.5091	0.8658	0.1967	0.184*	
C16	0.2739 (5)	0.9540 (3)	0.0911 (4)	0.127 (2)	
H16A	0.2339	0.9318	0.0463	0.190*	
H16B	0.3047	1.0182	0.0840	0.190*	
H16C	0.2119	0.9532	0.1306	0.190*	
C17	0.4848 (4)	0.8855 (3)	0.0467 (2)	0.0856 (11)	
H17A	0.4712	0.9373	0.0119	0.103*	
H17B	0.5723	0.8891	0.0656	0.103*	
C18	0.4599 (3)	0.7908 (2)	0.0114 (2)	0.0647 (9)	
C19	0.5074 (3)	0.7502 (3)	−0.0522 (2)	0.0731 (10)	
H19	0.5640	0.7840	−0.0824	0.088*	
C20	0.4699 (4)	0.6584 (3)	−0.0705 (2)	0.0833 (11)	
H20	0.5022	0.6299	−0.1132	0.100*	
C21	0.3851 (3)	0.6086 (3)	−0.0262 (2)	0.0729 (10)	
H21	0.3603	0.5469	−0.0395	0.088*	
C22	0.3368 (3)	0.6491 (2)	0.03737 (19)	0.0605 (8)	
C23	0.3733 (3)	0.7411 (2)	0.05565 (19)	0.0584 (8)	
C24	0.2899 (3)	0.5289 (2)	0.12496 (18)	0.0564 (8)	
C25	0.2109 (4)	0.4110 (3)	0.2117 (2)	0.0874 (12)	
H25A	0.2936	0.3798	0.2046	0.105*	
H25B	0.1439	0.3627	0.2084	0.105*	
C26	0.2072 (7)	0.4562 (6)	0.2855 (3)	0.166 (3)	
H26A	0.2746	0.5031	0.2890	0.249*	
H26B	0.2196	0.4081	0.3228	0.249*	
H26C	0.1250	0.4866	0.2926	0.249*	
H1	0.622 (4)	0.504 (2)	0.1267 (18)	0.056 (10)*	
H2	0.117 (4)	0.503 (3)	0.144 (2)	0.072 (11)*	

### Atomic displacement parameters (Å^2^)

**Table:**

	*U*^11^	*U*^22^	*U*^33^	*U*^12^	*U*^13^	*U*^23^
O1	0.0670 (16)	0.0924 (17)	0.0808 (17)	0.0327 (14)	0.0019 (13)	−0.0074 (14)
O2	0.0409 (12)	0.0715 (15)	0.144 (2)	−0.0031 (11)	−0.0087 (15)	0.0076 (15)
O3	0.0450 (12)	0.0685 (14)	0.1038 (18)	0.0029 (11)	0.0026 (12)	−0.0232 (13)
O4	0.0812 (16)	0.0653 (13)	0.0811 (16)	−0.0084 (13)	0.0173 (13)	0.0042 (12)
O5	0.0460 (11)	0.0603 (12)	0.1001 (18)	0.0058 (10)	0.0084 (12)	0.0259 (12)
O6	0.0417 (12)	0.0868 (16)	0.115 (2)	0.0025 (11)	−0.0052 (13)	0.0285 (16)
N1	0.0387 (15)	0.0758 (19)	0.098 (2)	0.0029 (14)	−0.0018 (15)	0.0000 (17)
N2	0.0449 (15)	0.0672 (16)	0.086 (2)	−0.0045 (14)	−0.0054 (14)	0.0208 (15)
C1	0.064 (2)	0.084 (2)	0.081 (2)	0.023 (2)	0.0069 (18)	−0.012 (2)
C2	0.091 (3)	0.090 (3)	0.143 (4)	0.013 (3)	0.032 (3)	−0.007 (3)
C3	0.122 (4)	0.151 (5)	0.107 (4)	0.045 (4)	−0.030 (3)	−0.049 (3)
C4	0.084 (3)	0.092 (3)	0.104 (3)	0.034 (2)	0.010 (2)	−0.003 (2)
C5	0.064 (2)	0.0597 (18)	0.094 (3)	0.0074 (17)	0.0040 (19)	−0.0047 (19)
C6	0.095 (3)	0.062 (2)	0.107 (3)	0.016 (2)	0.003 (3)	0.004 (2)
C7	0.110 (4)	0.072 (2)	0.102 (3)	0.010 (3)	0.013 (3)	0.008 (2)
C8	0.095 (3)	0.070 (2)	0.086 (3)	−0.006 (2)	0.019 (2)	−0.006 (2)
C9	0.0520 (18)	0.0592 (18)	0.093 (3)	−0.0028 (15)	0.0054 (18)	−0.0162 (18)
C10	0.0541 (19)	0.0673 (19)	0.077 (2)	0.0050 (16)	−0.0014 (17)	−0.0106 (18)
C11	0.0451 (17)	0.0670 (19)	0.072 (2)	−0.0005 (15)	0.0029 (15)	−0.0018 (17)
C12	0.063 (2)	0.082 (2)	0.135 (4)	0.007 (2)	0.000 (3)	0.029 (3)
C13	0.147 (5)	0.094 (3)	0.168 (5)	0.004 (4)	−0.023 (5)	−0.044 (3)
C14	0.073 (2)	0.0630 (19)	0.096 (3)	−0.0086 (18)	0.004 (2)	−0.0076 (19)
C15	0.124 (4)	0.136 (4)	0.108 (4)	−0.031 (4)	0.006 (3)	−0.024 (3)
C16	0.091 (3)	0.076 (3)	0.213 (6)	0.008 (3)	0.011 (4)	0.015 (3)
C17	0.084 (3)	0.077 (2)	0.096 (3)	−0.021 (2)	0.006 (2)	0.011 (2)
C18	0.0516 (18)	0.0676 (19)	0.075 (2)	−0.0058 (16)	−0.0013 (16)	0.0140 (18)
C19	0.0522 (19)	0.092 (3)	0.075 (2)	−0.0105 (19)	0.0040 (18)	0.012 (2)
C20	0.063 (2)	0.102 (3)	0.085 (3)	−0.004 (2)	0.015 (2)	−0.012 (2)
C21	0.058 (2)	0.069 (2)	0.092 (3)	−0.0068 (17)	0.0035 (19)	−0.002 (2)
C22	0.0447 (16)	0.0575 (17)	0.079 (2)	0.0017 (14)	0.0040 (16)	0.0155 (16)
C23	0.0476 (17)	0.0603 (17)	0.0673 (19)	−0.0004 (14)	0.0007 (15)	0.0105 (15)
C24	0.0436 (16)	0.0517 (15)	0.074 (2)	−0.0015 (13)	−0.0072 (15)	0.0029 (16)
C25	0.070 (2)	0.087 (3)	0.106 (3)	−0.003 (2)	0.000 (2)	0.037 (2)
C26	0.187 (7)	0.215 (7)	0.096 (4)	0.112 (6)	0.043 (4)	0.060 (4)

### Geometric parameters (Å, °)

**Table:**

O1—C10	1.356 (5)	C8—H8	0.9300
O1—C1	1.486 (4)	C9—C10	1.381 (5)
O2—C11	1.198 (4)	C12—C13	1.478 (7)
O3—C11	1.373 (4)	C12—H12A	0.9700
O3—C9	1.406 (4)	C12—H12B	0.9700
O4—C23	1.364 (4)	C13—H13A	0.9600
O4—C14	1.491 (4)	C13—H13B	0.9600
O5—C24	1.365 (4)	C13—H13C	0.9600
O5—C22	1.402 (4)	C14—C16	1.506 (6)
O6—C24	1.203 (4)	C14—C15	1.515 (6)
N1—C11	1.321 (4)	C14—C17	1.545 (6)
N1—C12	1.443 (5)	C15—H15A	0.9600
N1—H1	0.82 (4)	C15—H15B	0.9600
N2—C24	1.320 (4)	C15—H15C	0.9600
N2—C25	1.451 (5)	C16—H16A	0.9600
N2—H2	0.84 (4)	C16—H16B	0.9600
C1—C3	1.497 (6)	C16—H16C	0.9600
C1—C2	1.498 (6)	C17—C18	1.491 (5)
C1—C4	1.532 (6)	C17—H17A	0.9700
C2—H2A	0.9600	C17—H17B	0.9700
C2—H2B	0.9600	C18—C19	1.372 (5)
C2—H2C	0.9600	C18—C23	1.389 (4)
C3—H3A	0.9600	C19—C20	1.379 (6)
C3—H3B	0.9600	C19—H19	0.9300
C3—H3C	0.9600	C20—C21	1.377 (5)
C4—C5	1.506 (6)	C20—H20	0.9300
C4—H4A	0.9700	C21—C22	1.375 (5)
C4—H4B	0.9700	C21—H21	0.9300
C5—C6	1.376 (6)	C22—C23	1.379 (5)
C5—C10	1.389 (5)	C25—C26	1.475 (7)
C6—C7	1.364 (6)	C25—H25A	0.9700
C6—H6	0.9300	C25—H25B	0.9700
C7—C8	1.385 (6)	C26—H26A	0.9600
C7—H7	0.9300	C26—H26B	0.9600
C8—C9	1.379 (6)	C26—H26C	0.9600
			
C10—O1—C1	107.6 (3)	C12—C13—H13B	109.5
C11—O3—C9	118.0 (2)	H13A—C13—H13B	109.5
C23—O4—C14	107.3 (3)	C12—C13—H13C	109.5
C24—O5—C22	116.8 (2)	H13A—C13—H13C	109.5
C11—N1—C12	122.8 (3)	H13B—C13—H13C	109.5
C11—N1—H1	118 (2)	O4—C14—C16	106.5 (3)
C12—N1—H1	117 (2)	O4—C14—C15	106.0 (4)
C24—N2—C25	121.2 (3)	C16—C14—C15	112.8 (4)
C24—N2—H2	117 (3)	O4—C14—C17	105.4 (3)
C25—N2—H2	121 (3)	C16—C14—C17	111.2 (4)
O1—C1—C3	106.6 (3)	C15—C14—C17	114.2 (4)
O1—C1—C2	106.3 (3)	C14—C15—H15A	109.5
C3—C1—C2	114.3 (4)	C14—C15—H15B	109.5
O1—C1—C4	105.5 (3)	H15A—C15—H15B	109.5
C3—C1—C4	112.0 (4)	C14—C15—H15C	109.5
C2—C1—C4	111.4 (4)	H15A—C15—H15C	109.5
C1—C2—H2A	109.5	H15B—C15—H15C	109.5
C1—C2—H2B	109.5	C14—C16—H16A	109.5
H2A—C2—H2B	109.5	C14—C16—H16B	109.5
C1—C2—H2C	109.5	H16A—C16—H16B	109.5
H2A—C2—H2C	109.5	C14—C16—H16C	109.5
H2B—C2—H2C	109.5	H16A—C16—H16C	109.5
C1—C3—H3A	109.5	H16B—C16—H16C	109.5
C1—C3—H3B	109.5	C18—C17—C14	103.7 (3)
H3A—C3—H3B	109.5	C18—C17—H17A	111.0
C1—C3—H3C	109.5	C14—C17—H17A	111.0
H3A—C3—H3C	109.5	C18—C17—H17B	111.0
H3B—C3—H3C	109.5	C14—C17—H17B	111.0
C5—C4—C1	103.7 (3)	H17A—C17—H17B	109.0
C5—C4—H4A	111.0	C19—C18—C23	120.5 (3)
C1—C4—H4A	111.0	C19—C18—C17	131.5 (3)
C5—C4—H4B	111.0	C23—C18—C17	108.0 (3)
C1—C4—H4B	111.0	C18—C19—C20	118.9 (3)
H4A—C4—H4B	109.0	C18—C19—H19	120.6
C6—C5—C10	119.7 (4)	C20—C19—H19	120.6
C6—C5—C4	133.1 (3)	C21—C20—C19	120.7 (4)
C10—C5—C4	107.2 (3)	C21—C20—H20	119.7
C7—C6—C5	119.8 (4)	C19—C20—H20	119.7
C7—C6—H6	120.1	C22—C21—C20	120.7 (4)
C5—C6—H6	120.1	C22—C21—H21	119.7
C6—C7—C8	121.0 (4)	C20—C21—H21	119.7
C6—C7—H7	119.5	C21—C22—C23	118.9 (3)
C8—C7—H7	119.5	C21—C22—O5	120.6 (3)
C9—C8—C7	119.7 (4)	C23—C22—O5	120.4 (3)
C9—C8—H8	120.2	O4—C23—C22	125.4 (3)
C7—C8—H8	120.2	O4—C23—C18	114.3 (3)
C8—C9—C10	119.3 (3)	C22—C23—C18	120.3 (3)
C8—C9—O3	119.8 (3)	O6—C24—N2	126.8 (3)
C10—C9—O3	120.6 (3)	O6—C24—O5	123.6 (3)
O1—C10—C9	125.1 (3)	N2—C24—O5	109.6 (3)
O1—C10—C5	114.4 (3)	N2—C25—C26	110.6 (4)
C9—C10—C5	120.5 (3)	N2—C25—H25A	109.5
O2—C11—N1	127.4 (3)	C26—C25—H25A	109.5
O2—C11—O3	122.7 (3)	N2—C25—H25B	109.5
N1—C11—O3	109.9 (3)	C26—C25—H25B	109.5
N1—C12—C13	111.3 (4)	H25A—C25—H25B	108.1
N1—C12—H12A	109.4	C25—C26—H26A	109.5
C13—C12—H12A	109.4	C25—C26—H26B	109.5
N1—C12—H12B	109.4	H26A—C26—H26B	109.5
C13—C12—H12B	109.4	C25—C26—H26C	109.5
H12A—C12—H12B	108.0	H26A—C26—H26C	109.5
C12—C13—H13A	109.5	H26B—C26—H26C	109.5
			
C10—O1—C1—C3	131.3 (4)	C23—O4—C14—C16	107.5 (4)
C10—O1—C1—C2	−106.3 (4)	C23—O4—C14—C15	−132.1 (4)
C10—O1—C1—C4	12.1 (4)	C23—O4—C14—C17	−10.7 (4)
O1—C1—C4—C5	−12.4 (4)	O4—C14—C17—C18	11.4 (4)
C3—C1—C4—C5	−128.0 (4)	C16—C14—C17—C18	−103.6 (4)
C2—C1—C4—C5	102.6 (4)	C15—C14—C17—C18	127.4 (4)
C1—C4—C5—C6	−173.3 (4)	C14—C17—C18—C19	172.3 (4)
C1—C4—C5—C10	8.7 (5)	C14—C17—C18—C23	−8.4 (4)
C10—C5—C6—C7	1.2 (6)	C23—C18—C19—C20	−1.4 (5)
C4—C5—C6—C7	−176.6 (5)	C17—C18—C19—C20	177.9 (4)
C5—C6—C7—C8	0.5 (7)	C18—C19—C20—C21	0.7 (6)
C6—C7—C8—C9	−1.1 (7)	C19—C20—C21—C22	−0.4 (6)
C7—C8—C9—C10	−0.1 (6)	C20—C21—C22—C23	0.8 (5)
C7—C8—C9—O3	174.4 (3)	C20—C21—C22—O5	176.5 (3)
C11—O3—C9—C8	106.3 (4)	C24—O5—C22—C21	78.1 (4)
C11—O3—C9—C10	−79.3 (4)	C24—O5—C22—C23	−106.3 (3)
C1—O1—C10—C9	175.9 (3)	C14—O4—C23—C22	−174.8 (3)
C1—O1—C10—C5	−6.9 (4)	C14—O4—C23—C18	5.7 (4)
C8—C9—C10—O1	178.9 (4)	C21—C22—C23—O4	179.0 (3)
O3—C9—C10—O1	4.4 (5)	O5—C22—C23—O4	3.3 (5)
C8—C9—C10—C5	1.8 (5)	C21—C22—C23—C18	−1.6 (5)
O3—C9—C10—C5	−172.6 (3)	O5—C22—C23—C18	−177.2 (3)
C6—C5—C10—O1	−179.7 (4)	C19—C18—C23—O4	−178.6 (3)
C4—C5—C10—O1	−1.4 (5)	C17—C18—C23—O4	2.0 (4)
C6—C5—C10—C9	−2.4 (6)	C19—C18—C23—C22	1.9 (5)
C4—C5—C10—C9	175.9 (3)	C17—C18—C23—C22	−177.5 (3)
C12—N1—C11—O2	−6.4 (7)	C25—N2—C24—O6	9.2 (6)
C12—N1—C11—O3	173.9 (3)	C25—N2—C24—O5	−171.5 (3)
C9—O3—C11—O2	−2.7 (5)	C22—O5—C24—O6	7.3 (5)
C9—O3—C11—N1	176.9 (3)	C22—O5—C24—N2	−172.0 (3)
C11—N1—C12—C13	−96.9 (5)	C24—N2—C25—C26	89.0 (5)

### Hydrogen-bond geometry (Å, °)

**Table:**

*D*—H···*A*	*D*—H	H···*A*	*D*···*A*	*D*—H···*A*
N1—H1···O6	0.82 (4)	2.28 (4)	3.024 (4)	151 (3)
N2—H2···O2^i^	0.84 (4)	2.19 (4)	2.985 (4)	156 (3)
C19—H19···O5^ii^	0.93	2.48	3.269 (4)	143

Symmetry codes: (i) *x*−1, *y*, *z*; (ii) *x*+1/2, −*y*+3/2, −*z*.
